# Pressure-Induced Amorphization of Small Pore Zeolites—the Role of Cation-H_2_O Topology and Anti-glass Formation

**DOI:** 10.1038/srep15056

**Published:** 2015-10-12

**Authors:** Gil Chan Hwang, Tae Joo Shin, Douglas A. Blom, Thomas Vogt, Yongjae Lee

**Affiliations:** 1 Department of Earth System Sciences, Yonsei University, Seoul, 120749, Korea; 2 UNIST Central Research Facilities & School of Natural Science, Ulsan National Institute of Science and Technology (UNIST), Ulsan, 689798, Korea; 3 NanoCenter & Department of Chemistry and Biochemistry, University of South Carolina, Columbia, SC 29208, USA

## Abstract

Systematic studies of pressure-induced amorphization of natrolites (PIA) containing monovalent extra-framework cations (EFC) Li^+^, Na^+^, K^+^, Rb^+^, Cs^+^ allow us to assess the role of two different EFC-H_2_O configurations within the pores of a zeolite: one arrangement has H_2_O molecules (NAT_I_) and the other the EFC (NAT_II_) in closer proximity to the aluminosilicate framework. We show that NAT_I_ materials have a lower onset pressure of PIA than the NAT_II_ materials containing Rb and Cs as EFC. The onset pressure of amorphization (P_A_) of NAT_II_ materials increases linearly with the size of the EFC, whereas their initial bulk moduli (P1 phase) decrease linearly. Only Cs- and Rb-NAT reveal a phase separation into a dense form (P2 phase) under pressure. High-Angle Annular Dark Field Scanning Transmission Electron Microscopy (HAADF-STEM) imaging shows that after recovery from pressures near 25 and 20 GPa long-range ordered Rb-Rb and Cs-Cs correlations continue to be present over length scales up to 100 nm while short-range ordering of the aluminosilicate framework is significantly reduced—this opens a new way to form anti-glass structures.

Pressure-induced amorphization (PIA), the loss of long-range order under pressure, first discovered in ice^1^, is common among open framework structures such as zeolites and metal-organic frameworks (MOFs) and often attributed to softening of low-energy vibrations of the framework and local distortions^2^,^3^,^4^. The potential use of PIA to synthesize ‘ideal glasses’ has been put forward^1^,^2^. Assessing if and to what a degree materials truly lack long-range order and are amorphous is dependent on the experimental conditions: in the pressure-induced amorphization of anorthite (CaAl_2_Si_2_O_8_) X-ray Bragg reflections broaden and loose intensities at pressures lower than observed by Williams and Jeanloz using optical birefringence measurements^5^. We use X-ray powder diffraction and High Angle Annular Dark Field Scanning Transmission Electron Microscopy (HAADF STEM) imaging in real space to characterize the extent of long range order that continues to be present after the samples have been pressurized in the presence of silicone oil, a non-pore penetrating pressure-transmission fluid.

In large pore zeolites such as silicalite it has been shown that the incorporation of certain guest molecules such as Ar and CO_2_ delays PIA from pressures near 8 GPa up to 25 GPa and at the same time significantly increases the bulk modulus^6^. In AlPO_4_-54-xH_2_O water coordination to the Al^3+^ framework has been suggested to initiate PIA^7^. We present systematic studies of PIA in small pore zeolites with natrolite (NAT) frameworks. Our studies investigate the impact different EFC-H_2_O arrangements have on the onset pressure of PIA.

Na-NAT (Na_16_Al_16_Si_24_O_80_•16H_2_O) is a natural zeolite with small elliptic pores having a conjugate diameter of less than 4 Å. However, under pressure in water-containing fluids pressure-induced hydration occurs due to an expansion and pore-opening in this auxetic material^8^. This behavior was established in Li-, K-, Rb-NAT while Cs-NAT reveals a distinct pressure-induced phase transition without pressure-induced hydration^9^. Computational studies by Kremleva *et al.* using density functional theory were able to optimize the structures of Li^+^, Na^+^, K^+^, Rb^+^, Cs^+^ containing natrolites at ambient conditions and point to the importance of the EFC-H_2_O interactions and the strain energy of the aluminosilicate framework under pressure^10^.

## Results

### Structural changes under pressure

**Figure 1:**
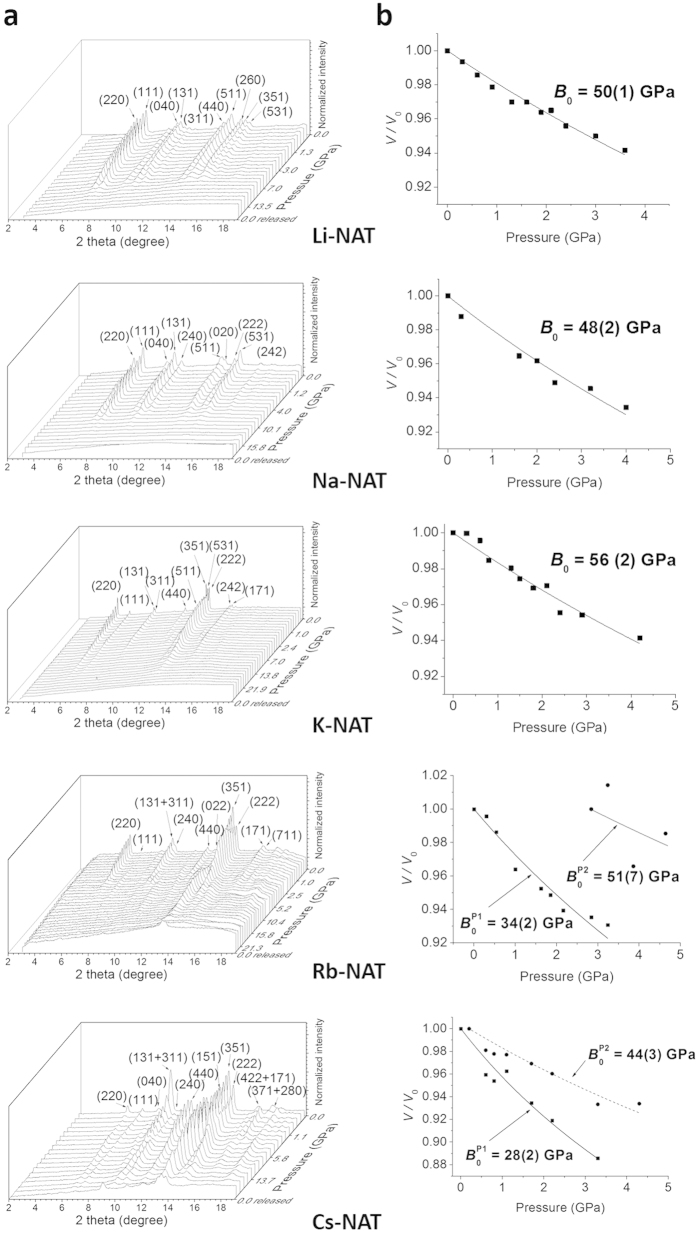
Pressure-dependent changes in (a) the XRD patterns of monovalent natrolites (NAT) using silicone oil as a pressure transmission medium. Note the phase separation in Rb-NAT and Cs-NAT, and (**b**) the unit cell volume and derived bulk moduli using the equation-of-state by Angel^22^,^23^ (see Supplementary Table S1).

When subjected to pressure in the presence of the non-pore penetrating pressure transmitting fluid silicone oil one observes gradual shifts of all diffraction peaks to higher 2-theta indicating normal compression of the unit cell lengths and volume (Fig. 1a and Supplementary Table S1). As silicone oil only transmits hydrostatic pressure up to near 5 GPa^11^ we only used data up to this pressure to determine bulk moduli. These values indicate that the bulk moduli of the NAT_I_ members Li-NAT and Na-NAT and the NAT_II_ members K-NAT and Rb-NAT (P2) and Cs-NAT(P2) are within error the same whereas the P(1) phases of K-NAT, Rb-NAT, and Cs-NAT are significantly different. (Figures 1b and 3a and Supplementary Table S2).

**Figure 2:**
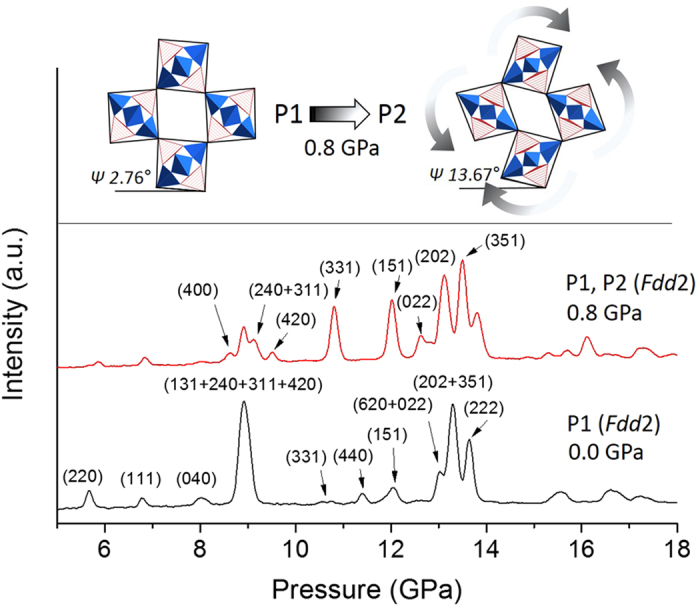
Framework models of P1 and P2 phases in Cs-NAT derived from laboratory-XRD data collected at 0.0 GPa and 0.8 GPa, respectively (Supplementary Table S3). The rotation angles of the chain (*Ψ*) increases from 2.7(1)° in P1 to 13.7(1)° in P2. The Cs-water distributions have not been considered due to the limited 2-theta range.

In Rb-NAT and Cs-NAT, as the frameworks are compressed in silicone oil two coexisting phases with smaller unit cell volumes are observed at 2.8 and 0.2 GPa (Figures 1 and 2), respectively. These phases were modeled to be isostructural to the ambient phases but with a more efficient EFC-H_2_O packing in the channels (Fig. 2 and Supplementary Table S3). Laboratory XRD data up to 18° 2-theta allowed us to estimate that the smaller volume P2 phases have chain rotation angles about 10° larger than the P1 phases which have approximately 100 Å^3^ larger unit cells (see Supplementary Tables S1 and S3). The bulk moduli of the dense phases (P2) with 51(7) GPa and 50(3) GPa for Rb-NAT (P2) and Cs-NAT (P2), respectively, are comparable to those of NAT_I_ phases while the P1 phases with larger unit cell volumes scale differently with the cation radius (see Supplementary Table S2). Further increase in pressure leads to PIA as observed by the broadening and weakening of the diffraction peaks (Fig. 1a and Supplementary Fig. S1).

### Distribution of EFCs in type-I and -II natrolites

**Figure 3:**
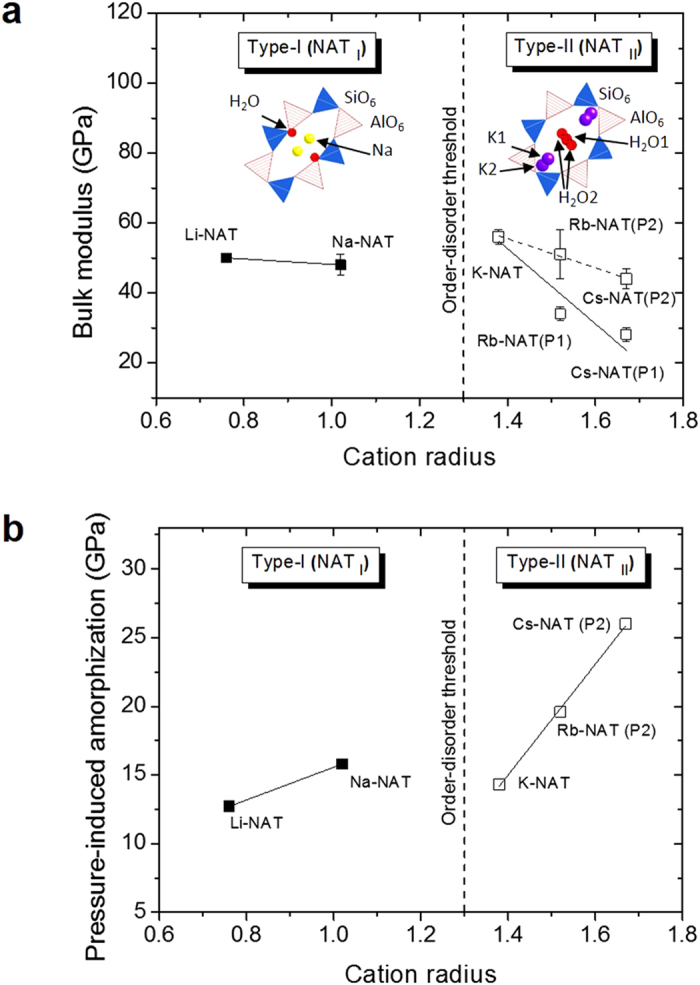
Bulk moduli and pressure-induced amorphization (a) Bulk moduli as a function of monovalent cation radius in natrolites. Note two coexisting phases for Rb-NAT and Cs-NAT. The bulk moduli of Li-NAT, Na-NAT, K-NAT, Rb-NAT(P2) and Cs-NAT(P2) are within error the same. (**b**) Onset pressure of pressure-induced amorphization as a function of cation radius in natrolites. The order-disorder threshold separates NAT_I_ and NAT_II_-type materials where the later has a disordered EFC-H_2_O substructure.

Kremleva established that the EFC-H_2_O interaction energies decrease with EFC size and can be grouped into type-I natrolites (NAT_I_), Li- and Na-NAT, where the H_2_O molecules interact strongly with the EFC and type-II natrolites (NAT_II_), K-, Rb- and Cs-NAT, where EFC-H_2_O interactions are weaker^12^. The structures of these two types have a distinct EFC-H_2_O topology: in NAT_I_, the H_2_O molecules are in close proximity to the aluminosilicate framework and Li^+^ and Na^+^ are found near the center of the pores, aligned along the *c*-axis alternately binding water molecules. In NAT_II_ containing K^+^, Rb^+^ and Cs^+^, the EFC and H_2_O molecules switch places and the H_2_O molecules are now located close to the center of the pores and the EFC near the aluminosilicate framework (see inset in Fig. 3a and Supplementary Fig. S2). In NAT_II_ systems the H_2_O-EFC chains are disordered and more than one isomer can exist. DFT calculations in the case of Cs-NAT reveal that the energy differences between different isomer structures are about 2 kJ mol^−1^ pointing to a very flat potential energy surface^11^. The strain energy of the natrolite framework varies significantly as a function of EFC size as the T-O-T (T = Al, Si) angle increases from 130° in Na-NAT to almost linear at 175° in Cs-NAT. The strain energy increases as the volume increases with the size of the EFC. As a consequence of this one needs to first exchange Na^+^ by K^+^ and then insert the larger EFC as the energies required to directly exchange Na^+^ by Rb^+^ and Cs^+^ are too big^13^.

### Bulk moduli, pressure-induced amorphization, and medium-range order after PIA

In our studies we defined the pressure at which the sum of all Bragg intensities *I* < 5% *I*_O_, *I*_O_ being the sum of all Bragg intensities at ambient conditions, as the onset pressure of PIA (P_A_) (Supplementary Fig. S3). We observe distinct changes of P_A_ with EFC size for both NAT_I_ and NAT_II_ (Fig. 3b): in the NAT_I_ materials, Li- and Na-NAT, which have stronger EFC-H_2_O interactions and the EFC located near the center of the pores, PIA occurs at 12.7(1) and 15.8(1) GPa, respectively. In earlier work Goryainov^14^ used a water free methanol-ethanol pressure transmitting medium and reported PIA in Na-NAT to be completed at 10 GPa, a value almost 6 GPa lower than what we found in silicone oil. This could point to an important influence of the pressure-transmitting medium in PIA as well as the fact that our pressure-conditions above 5 GPa are non-hydrostatic. In the NAT_II_ systems where K^+^, Rb^+^ and Cs^+^ are located closer to the zeolite framework and the H_2_O molecules near the center of the pores, a different PIA onset pressure dependence with the EFC size is observed. The onset pressures increase from 14.3(1) to 19.6(1) and 26.0(1) GPa, respectively (Fig. 3b). The peaks in the diffraction pattern of Rb- and Cs-NAT after PIA indicate that in these materials more than a short-range order persists, which is in contrast to what is found in Li-, Na- and K-NAT after PIA (Fig. 1a). After pressure release the recovered samples were heated to 250 °C over a period of 5 days and no recrystallization was found to occur (Supplementary Fig. S4). PIA in natrolites appears to be irreversible and we do not observe any significant changes in the XRD patterns over time.

**Figure 4:**
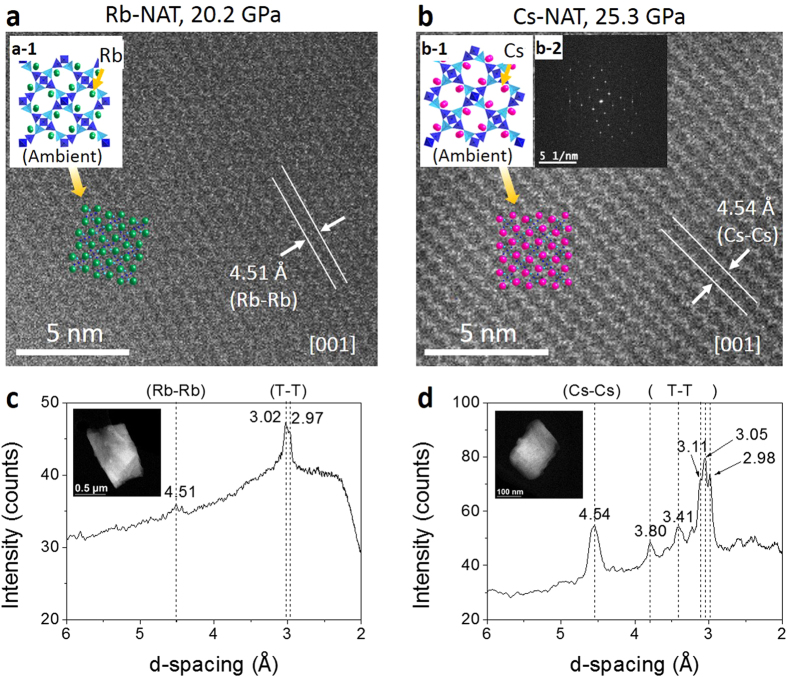
HAADF-STEM Z-contrast images of (a) Rb-NAT and (b) Cs-NAT recovered after being at pressures of 20.2 GPa and 25.3 GPa, respectively. Inset pictures show structural models, which are overlaid on the images to emphasize the retention of cation ordering. Second inset in b2) reveals Fourier transformation of real space image confirming long-range order of Cs-NAT. XRD patterns of (**c**) Rb-NAT and (**d**) Cs-NAT measured at 20.2 GPa and 25.3 GPa, respectively, are shown for comparison.

HAADF-STEM imaging using an aberration-corrected JEOL 2100F revealed that medium-range order up to 100 nm persists in both Rb- and Cs-NAT after PIA (Fig. 4). The contrast observed in the STEM images scales with approximately Z^2^, Z being the atomic number, and is predominantly due to the Rb- and Cs- cations. The inset in Fig. 4b-2 shows the Fourier transform of the STEM image of Cs-NAT after PIA and clearly indicating the presence of significant order beyond short-range correlations. In both Cs- and Rb-NAT samples both T-T (T = Al, Si) and EFC-EFC correlations are persistent as indicated by the HAADF-STEM real space images and intensities at appropriated-spacing in the XRD pattern. Cs-NAT despite having a higher onset pressure for PIA has retained a larger degree of order. Both samples displayed rapid beam damage when we attempted to image at higher resolution. This is not surprising as the presence of water within the zeolite cages has been shown to lead to radiolysis under electron beam irradiation which will be the initial key damage mechanism^15^,^16^. However, our real-space images clearly show that in Cs-NAT translational symmetry exists over length scales in the 100 nm range.

## Discussion

Our experiments unequivocally show that materials with the same aluminosilicate framework but different H_2_O-EFC topologies have different onset pressures of PIA and linear dependencies on cation size. Furthermore, the P1 phases of the NAT_II_ materials Rb-NAT and Cs-NAT have higher bulk compressibilities, both transform to two denser phases, and subsequently amorphize at higher pressures than NAT_I_ ones. We suggest that the closer proximity of water to the aluminosilicate framework in NAT_I_ materials appears to promote coordination with Al^3+^ which has been proposed as one mechanism for PIA in large pore materials^7^. The persistence of long-range order in Cs- and Rb-NAT after pressure release observed in X-ray powder data and HAADF-STEM images suggests an anti-glass structure. This structural model where translational symmetry is preserved over larger length scales while being disrupted at shorter ones was originally forwarded by Troemel for tellurium-oxide-based systems^17^. Recent structural work by Masson *et al.* on Bi_2_Te_4_O_11_ makes a case for the existence of such disorder^18^. Redfern argued that in metamict zircon (ZrSiO_4_) such an anti-glass structure is generated as the result of the α-decay of uranium and thorium located on Zr sites, which destroys local short-range order and over time forms areas of highly disordered material that separate ordered regions which remain registered over larger length scales^19^. The structure stabilizing role of EFC cations is well established in zeolite chemistry and the collapse of the framework structure around them due to the changes in T-O-T angles does not have to destroy their long range order. Our in-situ high-pressure powder diffraction experiments and the large strain energy found in DFT calculations of Cs- and Rb-NAT under pressure suggests a local disordering of the framework structure^10^. Subsequently the EFC are trapped and can no longer readily migrate to neighboring pores. This confinement within a disordered microporous aluminosilicate matrix can still ‘trap’ long-range order and explain the persistence of cation-cation correlations at higher q observed in both Rb- and Cs-NAT at high pressures as well as after pressure release.

## Methods

### Sample preparation

Ion-exchange procedures and characterizations of Na-NAT, Li-NAT, K-NAT, Rb-NAT, and Cs-NAT are reported in detail in Lee’s papers^13^,^20^. The procedures of ion-exchange was made by 2 steps. First of all, K-natrolite (K-NAT) was prepared using a 4 M KNO_3_ (ACS reagent grade from Sigma-Aldrich) solution and a grinded natrolite mineral (ideally Na_16_Al_16_Si_24_O_80_•16H_2_O, San Juan, Argentina) in a 100:1 weight ratio. The mixture was stirred at 80 °C in a closed system for 24 hours. The solid was separated from the solution by washing method using membrane filter (cellulose acetate 0.45 μm, Advantec). The dried powder was used for the third exchange cycles in the same conditions. The final product was air-dried, and from the elemental analysis (Jarrell-Ash Polyscan 61E Inductively Coupled Plasma), over 99% K-exchange was confirmed. Second, Li-natrolite (Li-NAT), Rb-natrolite (Rb-NAT) and Cs-natrolite (Cs-NAT) were then prepared using K-NAT as a starting material. Total three cycles of exchange were performed as for the K-exchange procedures, and nearly full Rb- and Cs-exchanges were confirmed, quantitatively, by SEM EDS method (the residual sodium and potassium ions were detected by only very small amounts, i.e., <0.1%). Li-, Rb-, and Cs-NAT were not directly exchanged cations from natrolite mineral (Na-NAT).

### High-pressure experiments

We used symmetric-type diamond anvil cell (DAC) with culet size of 400 μm. Gasket material used was stainless steel (T301) with about 120 μm indentation thickness and about 210 μm diameter sample hole made via μ-EDM (micro-electric discharging machine, Hylozoic products). Pressure at the sample was measured from ruby R1 fluorescence line using custom made μ-Raman system composed of Acton SP2500 spectrometer and PIXIS100 CCD detector (Princeton Instruments). Green laser of 532.3 nm in wavelength (SpectraPhysics/Newport Excelsior DPSS) was operated at 150 mW as an excitation source and collimated to ~20 μm via microscope system (Olympus BX41 plus ×20 Mitutoyo lens). Pressure was calculated from P (GPa) = 19.04/7.665[(1 + (Δλ/λ_0_))^7.665^−1]^21^.

### Temperature- and time-dependent experiments

Recovered gasket samples (Li-, Na-, K-, Rb-, Cs-NAT) were probed using laboratory XRD as a function of temperature- and time. Gaskets were heated inside a furnace (WiseTherm FH) at 50 °C, 100 °C, 150 °C, 200 °C and 250 °C over 5 days.

### X-ray diffraction

X-ray diffraction was performed using MicroMax-007HF (Rigaku Corp.) equipped with rotating anode Mo-Kα (λ = 0.7093 Å) with multilayer optics (VariMax-Mo, Rigaku) and imaging plate (IP) detector (R-axis IV^++^, Rigaku). X-ray generator was operated at 1.2 KW (50 kV, 24 mA), and X-ray beam size was reduced to 100 μm using pin-hole collimator. Sample to detector distance was about 120 mm, and typical exposure time was about 10 min. IPanalyzer v3.551 and Crystalclear v2.0 software were used to calibrate and process the 2D images. In the case of K-NAT, data were measured using synchrotron radiation at beamline 9A of Pohang light source (PLS). Experimental parameters were 20.07 KeV X-ray (λ = 0.6178 Å), 100 μm beam size, 15 sec of exposure time, and CCD detector (Rayonix SX165, 80 μm in pixel size) with S-to-D of 218.577 mm.

### HAADF-STEM measurement

High-Angle Annular Dark Field Scanning Transmission Electron Microscopy (HAADF STEM) was performed using a JEOL 2100F 200 kV FEG-STEM/TEM equipped with a CEOS Cs corrector on the illumination system to image the recovered Rb- and Cs-NAT. The geometrical aberrations were measured and controlled to provide less than a π/4 phase shift of the incoming electron wave over the probe-defining aperture of 17.5 mrad. High angle annular dark-field (HAADF) STEM images were acquired on a Fischione Model 3000 HAADF detector with a camera length such that the inner cut-off angle of the detector was 75 mrad. The scanning acquisition was synchronized to the 60 Hz AC electrical power to minimize 60 Hz noise in the images, and a pixel dwell time of 24 μs was used.

## Additional Information

**How to cite this article**: Hwang, G. C. *et al.* Pressure-Induced Amorphization of Small Pore Zeolites—the Role of Cation-H_2_O Topology and Anti-glass Formation. *Sci. Rep.* **5**, 15056; doi: 10.1038/srep15056 (2015).

## Supplementary Material

Supplementary Information
