# Supplementary material for: Pressure-Induced Amorphization of Small Pore Zeolites—the Role of Cation-H2O Topology and Anti-glass Formation
Source: Sci Rep. 2015 Oct 12;5:15056. doi: 10.1038/srep15056 (PMC4601026; doi:10.1038/srep15056)
Supplement: Supplementary Information [file srep15056-s1.pdf]

Supplementary Information

Pressure-Induced Amorphization of Small Pore Zeolites – the Role of Cation-Water Topology and Anti-glass Formation

Gil Chan Hwang[1], Tae Joo Shin[2], Douglas A. Blom[3], Thomas Vogt[3], and Yongjae Lee[1]

[1]Department of Earth System Sciences, Yonsei University, Seoul, 120749, Korea

[2]Pohang Accelerator Laboratory, Pohang University of Science and Technology (POSTECH), Pohang, 790784, Korea

[3]NanoCenter & Department of Chemistry and Biochemistry, University of South Carolina, Columbia, SC 29208, USA

Corresponding Author

Department of Earth System Sciences, Yonsei University, Seoul, 120749, Korea

Email) yongjaelee@yonsei.ac.kr

Office) +82-2-2123-5667

Fax) +82-2-2123-8169

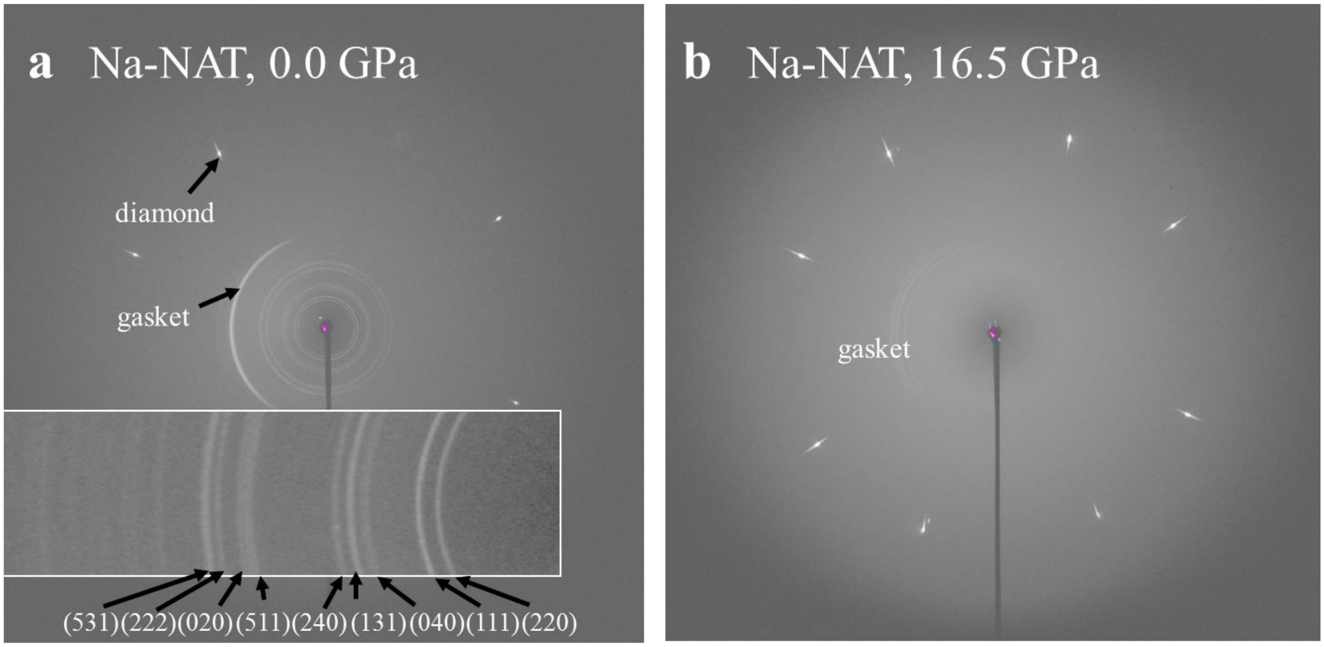

Supplementary Figure S1. Typical IP images before and after pressure-induced amorphization. (a) Na-NAT at ambient conditions in DAC and (b) its amorphous phase at 16.5 GPa under silicone oil as pressure medium.

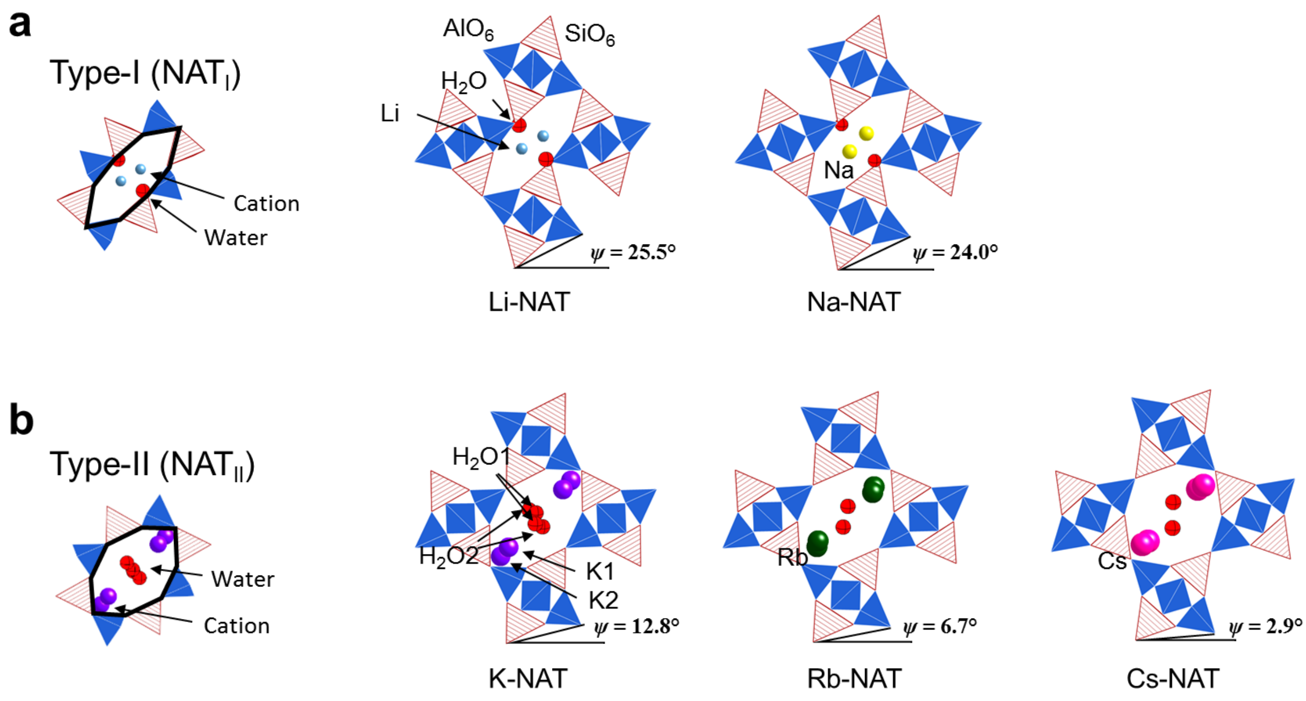

Supplementary Figure S2. Division of Type-I and –II natrolites by cation and water position at ambient conditions. (a**)** Type-I is Li-NAT and Na-NAT and (b**)** Type-II is K-NAT, Rb-NAT and Cs-NAT[13,20].

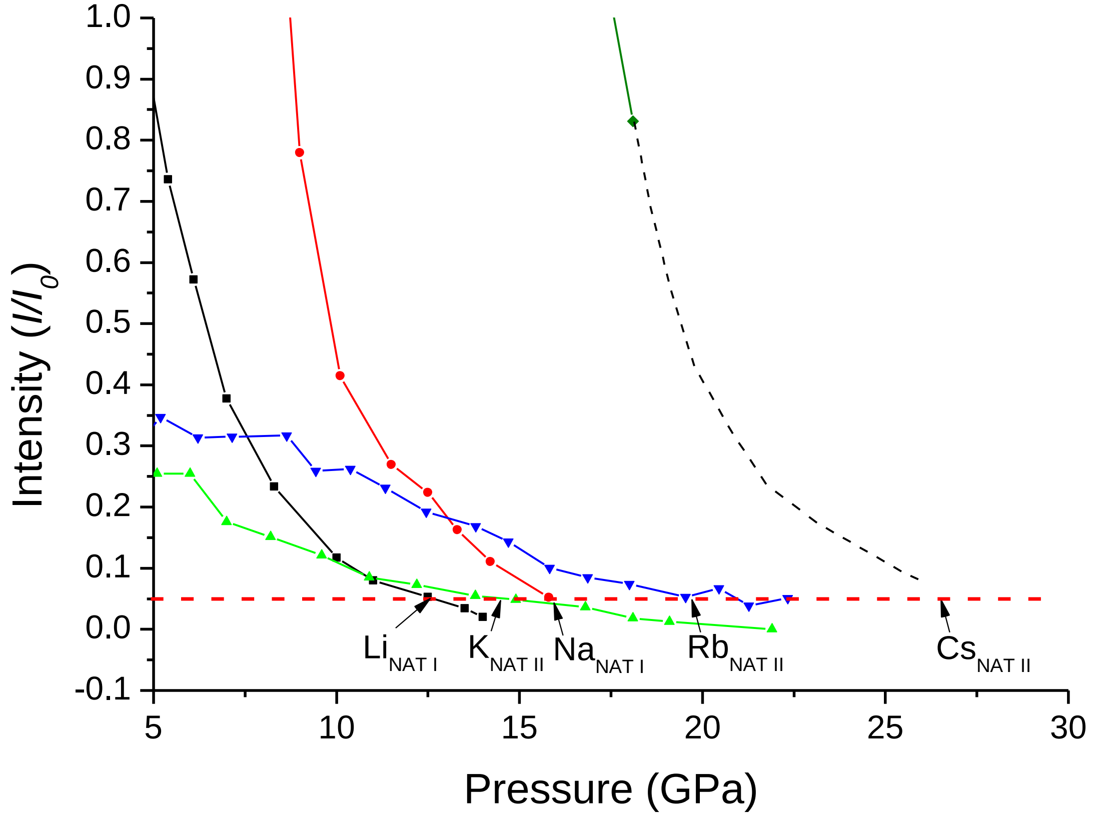

Supplementary Figure S3. Pressure-induced changes in the diffraction peak intensity for ion-exchanged natrolites. For Li-, Na- and K-NAT, (220) reflection was used, and for Rb- and Cs-NAT (351) and (022) reflections were used. The pressure at which I < 95% I_0 (horizontal dotted line) is defined as the onset of amorphization.

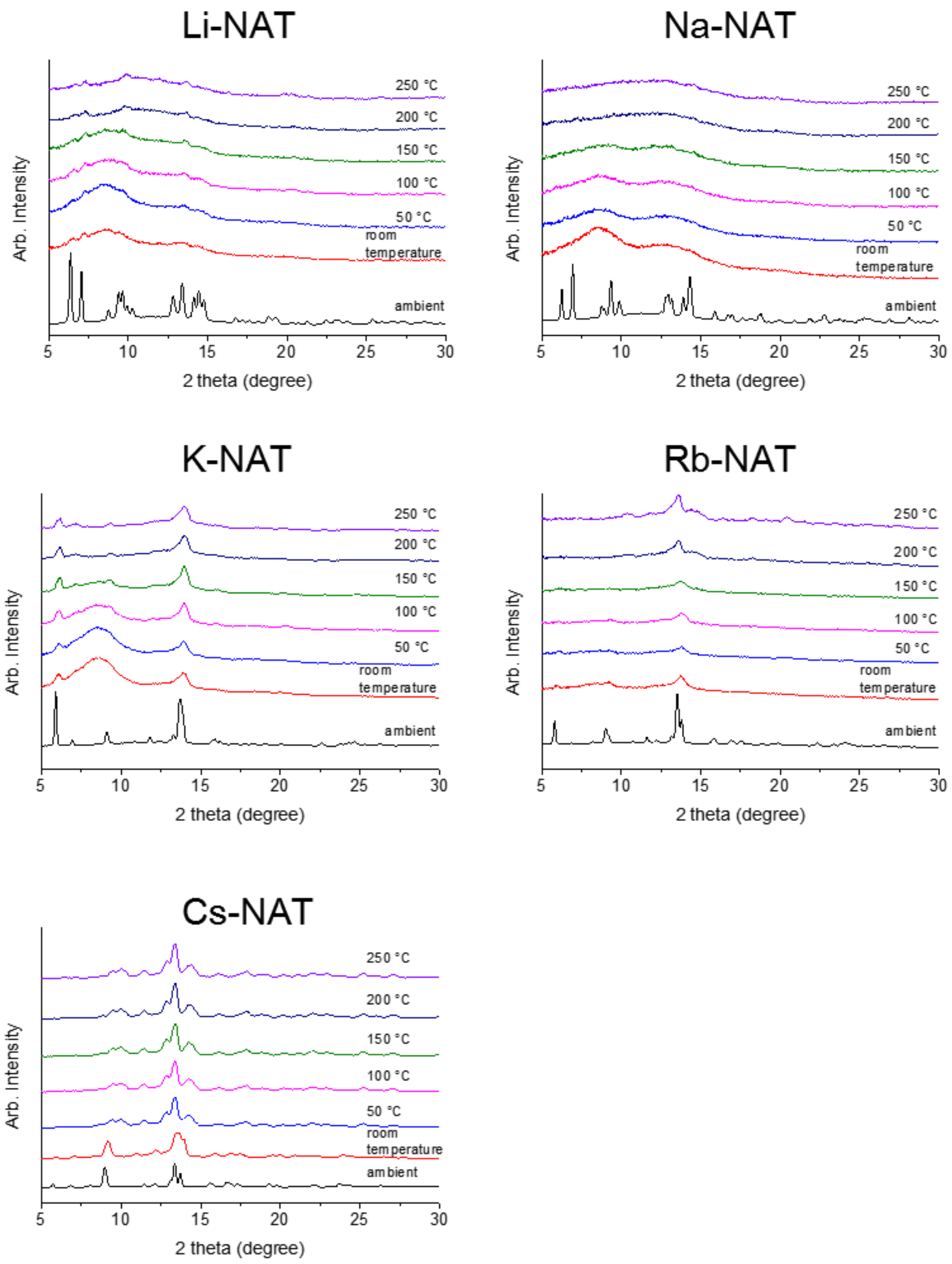

Supplementary Figure S4. Temperature-dependent variations of the XRD patterns of the ion-exchanged natrolites recovered from ca. 20 GPa under silicone oil pressure medium. The ambient pattern in the bottom represents XRD data before compression. It took 5 days from the first measurement at ambient condition after recovery to the last measurement after 250 ℃ heating.

Supplementary Table S1. Pressure-dependent variations of the unit cell parameters and volume of monovalent cation-exchanged natrolites.

P (GPa)	*a* (Å)	*b* (Å)	*c* (Å)	*V* (Å^3)	*P* (GPa)	*a* (Å)	*b* (Å)	*c* (Å)	*V* (Å^3)	Phase
**Li-NAT**, Compression ratio % (*a*:*b*:*c*:*V*, up to 4.1 GPa) = 4.6:0.9:2.2:7.6					**Rb-NAT**, Compression ratio % (*a*:*b*:*c*:*V*, up to 4.0 GPa) = 3.8:2.3:1.1:7.1					
0.0	17.629(2)	18.528(2)	6.521(0)	2129.8(3)	0.0	19.958(1)	19.903(2)	6.558(0)	2604.8(2)	
0.3	17.574(3)	18.587(3)	6.479(1)	2116.2(6)	0.3	19.708(2)	20.071(1)	6.557(0)	2593.6(2)	
0.6	17.473(2)	18.588(2)	6.464(0)	2099.2(4)	0.54	19.710(1)	19.892(2)	6.553(0)	2569.0(2)	
0.9	17.423(2)	18.535(2)	6.455(0)	2084.5(2)	0.84	19.723(1)	19.924(2)	6.581(0)	2586.0(2)	
1.3	17.337(2)	18.491(2)	6.443(0)	2065.6(3)	1.0	19.196(1)	19.931(1)	6.563(1)	2510.9(2)	
1.6	17.306(2)	18.512(3)	6.447(0)	2065.5(4)	1.23	19.186(1)	19.841(1)	6.509(1)	2477.6(2)	
2.1	17.259(2)	18.481(3)	6.437(0)	2053.1(3)	1.62	19.224(1)	19.754(1)	6.533(1)	2480.9(2)	
2.4	17.157(2)	18.466(2)	6.426(0)	2036.0(3)	1.85	19.256(3)	19.698(3)	6.514(1)	2470.8(7)	
3.0	17.099(2)	18.441(3)	6.416(0)	2023.2(3)	2.16	19.282(2)	19.589(2)	6.478(1)	2446.8(3)	
3.6	16.974(3)	18.430(2)	6.411(0)	2005.7(3)	2.47	19.540(4)	19.623(8)	6.500(1)	2492.2(14)	
4.1	16.810(2)	18.365(2)	6.374(0)	1967.9(4)	2.84	19.604(4)	19.203(5)	6.472(1)	2436.3(13)	P1
5.4	16.585(3)	18.325(2)	6.343(1)	1927.8(4)		19.406(6)	17.805(6)	6.485(1)	2240.5(13)	P2
6.1	16.420(3)	18.392(2)	6.329(0)	1911.3(4)	3.24	19.187(3)	19.568(3)	6.457(1)	2424.4(5)	P1
7.0	16.182(4)	18.491(4)	6.323(1)	1892.1(8)		17.964(3)	19.453(2)	6.504(2)	2272.7(5)	P2
8.3	15.603(8)	18.327(12)	6.212(3)	1776.4(22)	3.86	-	-	-	-	-
**Na-NAT**, Compression ratio % (*a*:*b*:*c*:*V*, up to 4.0 GPa) = 3.3:1.9:1.5:6.6						17.638(12)	19.300(13)	6.357(5)	2163.9(29)	P2
0.0	18.469(1)	18.697(2)	6.631(1)	2281.7(3)	4.64	-	-	-	-	-
0.3	18.387(2)	18.614(2)	6.608(0)	2261.7(2)		17.685(14)	19.281(11)	6.475(4)	2207.8(41)	P2
0.5	18.286(1)	18.498(1)	6.553(0)	2216.5(2)	5.19	18.642(5)	20.674(9)	6.616(2)	2549.8(14)	P1
0.9	18.240(3)	18.476(2)	6.581(1)	2217.8(3)		17.919(4)	19.422(3)	6.464(2)	2249.7(5)	P2
1.2	18.245(1)	18.471(1)	6.546(0)	2206.1(2)	6.21	18.771(10)	20.768(6)	6.574(2)	2563.0(15)	P1
1.6	18.253(1)	18.476(1)	6.549(0)	2208.8(3)		17.934(7)	19.379(5)	6.468(2)	2247.8(6)	P2
2.0	18.141(1)	18.488(1)	6.567(0)	2202.4(2)	7.15	18.572(11)	21.373(6)	6.682(2)	2652.3(16)	P1
2.4	18.050(1)	18.426(1)	6.533(0)	2172.9(1)		18.120(2)	17.895(8)	6.441(1)	2088.5(9)	P2
3.2	17.994(1)	18.405(1)	6.537(0)	2164.8(2)	**Cs-NAT**, Compression ratio % (*a*:*b*:*c*:*V*, up to 4.3 GPa) = 7.3:2.3:0.6:10.0					
4.0	17.852(1)	18.342(1)	6.533(0)	2139.2(2)	0.0	19.928(1)	20.251(1)	6.548(0)	2642.5(2)	
5.3	17.770(1)	18.249(1)	6.495(0)	2106.3(2)	0.2	19.927(3)	20.338(1)	6.546(1)	2653.2(5)	P1
6.1	17.597(1)	18.077(1)	6.464(0)	2056.2(2)		18.931(1)	20.424(1)	6.573(0)	2541.5(1)	P2
8.0	17.485(3)	17.999(3)	6.445(1)	2028.1(5)	0.6	19.000(1)	20.315(1)	6.595(0)	2545.4(1)	P1
9.0	17.284(7)	17.974(9)	6.458(3)	2006.1(25)		18.854(1)	20.255(1)	6.529(0)	2493.3(2)	P2
10.1	17.485(3)	17.794(5)	6.465(1)	2011.3(6)	0.8	18.972(1)	20.195(1)	6.577(0)	2520.1(2)	P1
11.5	17.117(4)	18.181(4)	6.521(1)	2029.2(7)		18.836(1)	20.213(1)	6.527(0)	2485.1(3)	P2
**K-NAT**, Compression ratio % (*a*:*b*:*c*:*V*, up to 4.2 GPa) = 2.4:2.7:0.9:5.9					1.1	18.986(1)	20.385(2)	6.579(0)	2546.4(3)	P1
0.0	19.356(0)	19.827(0)	6.499(0)	2493.9(1)		18.923(1)	20.0915(9)	6.531(0)	2483.2(2)	P2
0.3	19.338(1)	19.810(1)	6.508(0)	2493.2(2)	1.7	18.948(1)	20.013(1)	6.505(1)	2466.9(3)	P1
0.6	19.304(1)	19.792(1)	6.499(0)	2483.1(1)		18.818(1)	19.987(1)	6.550(0)	2463.4(2)	P2

0.8	19.155(2)	19.742(2)	6.493(0)	2455.3(4)	2.2	18.768(1)	20.072(1)	6.536(0)	2462.2(3)	P1
1.0	19.014(1)	19.717(2)	6.482(0)	2430.1(4)		18.702(1)	19.896(1)	6.559(0)	2440.6(2)	P2
1.3	19.046(1)	19.736(1)	6.504(0)	2444.8(2)	3.3	18.832(1)	19.526(1)	6.561(1)	2412.5(4)	P1
1.5	19.009(1)	19.666(1)	6.499(0)	2429.8(2)		18.472(2)	19.749(1)	6.503(0)	2372.1(3)	P2
1.8	18.992(1)	19.601(1)	6.493(0)	2417.0(2)	4.3	18.469(2)	19.770(1)	6.510(1)	2377.0(5)	P1
2.1	19.013(1)	19.590(1)	6.498(0)	2420.3(2)		18.800(1)	19.204(2)	6.574(0)	2373.6(3)	P2
2.4	18.933(3)	19.461(1)	6.466(1)	2382.5(10)	5.8	18.511(3)	18.871(3)	6.564(1)	2292.7(9)	
2.9	18.967(1)	19.408(2)	6.464(1)	2379.6(4)	6.9	18.356(2)	18.746(1)	6.564(0)	2258.7(3)	
4.2	18.897(2)	19.294(2)	6.439(1)	2347.5(4)	8.7	17.754(3)	18.462(3)	6.545(1)	2145.1(10)	
5.1	18.827(2)	19.188(2)	6.410(1)	2315.7(4)	10.1	17.760(1)	18.531(1)	6.568(0)	2161.6(3)	
6.0	18.814(1)	19.202(2)	6.409(1)	2315.5(4)	12.2	17.873(2)	18.571(1)	6.530(0)	2167.5(2)	
7.0	18.733(2)	19.128(3)	6.379(1)	2285.8(5)	13.7	17.724(4)	18.370(3)	6.531(1)	2126.5(5)	
8.2	18.648(5)	18.926(12)	6.333(1)	2235.0(14)	14.9	17.704(4)	18.351(3)	6.532(1)	2122.0(8)	
9.6	18.659(4)	18.969(12)	6.339(2)	2243.8(17)	16.5	17.697(5)	18.339(4)	6.505(1)	2111.3(9)	
10.2	18.458(4)	18.846(4)	6.301(2)	2192.0(8)	18.1	17.530(6)	18.101(4)	6.528(1)	2071.5(9)	
12.4	18.389(6)	18.856(9)	6.300(2)	2184.4(5)						
13.8	18.391(3)	18.864(4)	6.316(2)	2191.1(8)						
14.9	18.460(5)	18.910(8)	6.344(3)	2214.5(14)						
16.8	18.385(11)	18.824(14)	6.356(6)	2199.6(20)						
18.1	18.291(5)	18.881(7)	6.358(3)	2195.9(13)						
19.1	18.313(15)	18.977(16)	6.375(3)	2215.6(16)						

Supplementary Table S2. Bulk moduli (K_0) of the monovalent catioin-exchanged natrolites compressed using silicone oil as pressure medium. EOS-fit by R.J. Angel was used using K_0' fixed at 4.0 [22].

NAT series	K_0 (GPa)	*P* range (GPa)	Phase
Li	50(1)	0.0-3.6	
Na	48(2)	0.0-4.0	
K	56(2)	0.0-4.2	
Rb	34(2)	0.0-3.2	P1
	51(7)	2.8-4.6	P2
Cs	28(2)	0.0-3.3	P1
	44(3)	0.2-4.3	P2

Supplementary Table S3. Rietveld refinement model of the P2 phase in Cs-NAT at 0.8 GPa.

Phase			P2			
Space group			***F**dd2*			
*wR*p(%)			3.57			
Cell parameters			*a* (Å)	*b* (Å)	*c* (Å)	*V* (Å ^{3})
			18.811(4)	20.186(3)	6.547(1)	2486(1)
Atom	Site	*Occ.*	*x*	*y*	*z*	*Uiso*
Si(1)	8a	1	0	0	0	0.023
Si(2)	16b	1	0.1391(6)	0.2031(6)	0.589(2)	0.023
Al(1)	16b	1	0.034(1)	0.0751(1)	0.569(2)	0.023
O(1)	16b	1	0.037(1)	0.039(1)	0.812(2)	0.023
O(2)	16b	1	0.0757(5)	0.1517(6)	0.536(5)	0.023
O(3)	16b	1	0.093(1)	0.0259(0)	0.431(6)	0.023
O(4)	16b	1	0.2013(9)	0.1614(7)	0.703(3)	0.023
O(5)	16b	1	0.2001(0)	0.2137(9)	0.417(2)	0.023
OW	16b	1	0.361(1)	0.141(1)	0.267(5)	0.023
Cs(1)	16b	0.49	0.3084(3)	0.0512(3)	0.329(3)	0.023
Cs(2)	16b	0.43	0.2205(4)	0.0699(3)	0.329(3)	0.023

* Constrains were used for TO_4 bonding distances (1.62 Å for Si-O and 1.75 Å for Al-O) using GSAS software[23]. U_{iso} values and Cs occupancy were fixed following the model by Seoung et al[9].
